# Surgical Guides (Patient-Specific Instruments) for Pediatric Tibial Bone Sarcoma Resection and Allograft Reconstruction

**DOI:** 10.1155/2013/787653

**Published:** 2013-03-04

**Authors:** Laura Bellanova, Laurent Paul, Pierre-Louis Docquier

**Affiliations:** ^1^Computer-Assisted Robotic Surgery (CARS), Institut de Recherche Experimentale et Clinique (IREC), Tour Pasteur +4, Avenue Mounier 53, 1200 Brussels, Belgium; ^2^Department of Orthopaedic Surgery, Cliniques Universitaires Saint-Luc, (Université Catholique de Louvain), Avenue Hippocrate 10, 1200 Brussels, Belgium

## Abstract

To achieve local control of malignant pediatric bone tumors and to provide satisfactory oncological results, adequate resection margins are mandatory. The local recurrence rate is directly related to inappropriate excision margins. The present study describes a method for decreasing the resection margin width and ensuring that the margins are adequate. This method was developed in the tibia, which is a common site for the most frequent primary bone sarcomas in children. Magnetic resonance imaging (MRI) and computerized tomography (CT) were used for preoperative planning to define the cutting planes for the tumors: each tumor was segmented on MRI, and the volume of the tumor was coregistered with CT. After preoperative planning, a surgical guide (patient-specific instrument) that was fitted to a unique position on the tibia was manufactured by rapid prototyping. A second instrument was manufactured to adjust the bone allograft to fit the resection gap accurately. Pathologic evaluation of the resected specimens showed tumor-free resection margins in all four cases. The technologies described in this paper may improve the surgical accuracy and patient safety in surgical oncology. In addition, these techniques may decrease operating time and allow for reconstruction with a well-matched allograft to obtain stable osteosynthesis.

## 1. Introduction

The tibia is a common site for the most frequent malignant primary bone tumors in children, osteosarcoma (tibia is affected in 27% of cases), and Ewing's sarcoma (8% of cases) [1, 2]. Improvements in diagnosis and therapeutic techniques have increased interest in limb-salvage surgery. However, several studies have suggested that limb-salvage surgery may increase local recurrence in the case of inappropriate excision margins [3]. To achieve local control of disease and to improve oncological results, wide resection margins are mandatory. However, a wide surgical excision results in a large residual bone defect that requires restoration [4]. We propose a new technique to decrease the width of the excision margins with patient-specific instruments (PSIs). In this method, the resection is carefully planned prior to surgery, based on magnetic resonance imaging (MRI) and computed tomography (CT), which are used to define the trajectories of the resection and create PSIs.

The function of the reconstructed limb is of major interest, especially in young and physically active patients who place high demands on their limbs. The limb reconstruction must also be durable because life expectancy for many of these patients is several decades [4]. Limb reconstruction for such large defects can be performed by various techniques, including endoprosthetic reconstruction, osteoarticular allografting, vascularized autografting, bone transport with distraction osteogenesis, or reimplantation of the tumor-bearing bone segment after the devitalization of the tumor cells (by heating, freezing, or extracorporeal irradiation) [5].

Massive bone allografting presents several drawbacks: relatively long rehabilitation due to immobilization and partial loss of weight bearing, difficulties in obtaining size-matched allografts for small patients, an absence of expandability, and a relatively high incidence of complications [6]. Despite these drawbacks, allografting offers several advantages, including the ability to reattach the ligamentous and tendinous structures of the host to the graft. An accurate reconstruction of the soft-tissue attachments at the host-allograft junction can lead to improved results [7]. Other advantages include the biologic incorporation (at least partial) of the graft and the preservation of the joint, the juxta-articular bone, and the growth plates [8]. These advantages make massive bone allografting convenient for intercalary, osteoarticular, and arthrodesis operations, as well as for allograft-prosthetic composite reconstruction in an extra-articular resection. In fact, bone allografting is the most common option for intercalary reconstruction, with a survival rate as high as 75% to 89% at 10 years [6, 9–12].

The concept of using a patient-specific template was introduced in the 1990s by Radermacher et al. [13] for pedicle screw placement, total knee arthroplasty, decompression of cervical spine, and triple osteotomy of the pelvis. They performed CT-based preoperative planning and conceived a template to fit the bone surface. The template was manufactured by milling because rapid prototyping was not yet well developed and was far more expensive. Later, Salako et al. used guided intrapedicular screws to install instrumentation on the spine. This mechanism allowed the surgeon to drill in the optimal direction, and it decreased the rate of screw misplacement [14]. In maxillofacial reconstruction surgery, the use of a PSI to guide the osteotomies allowed the surgeon to avoid important structures, such as dental nerves and components of the vascular anatomy. This concept was used by Leiggener et al. [15] for mandible reconstruction with a free fibula osseous flap. Using CT angiography, the authors manufactured a guide by rapid prototyping (SLS). They performed a complex mandible reconstruction with this method, choosing the best sites on the donor (fibula) and recipient (mandible) with regard to function, aesthetics, and blood supply. Modabber et al. [16] concluded that this technique significantly decreased shaping time during surgery and will likely impact the survival of the flap. Several authors have used a patient-specific template technique to treat other conditions, such as a cubitus varus deformity, a malunited forearm fracture, and a distal-radial fracture combined with tibial deformities. After preoperative planning, a rapid-prototyped model was manufactured to correct each deformity. This technique improves the accuracy and ease of the surgical act [17, 18].

In the present paper, we describe the use of PSIs for resecting aggressive tibial sarcomas and reconstructing the anatomy with an intercalary or osteoarticular allograft. First, we detail the preoperative planning process. Next, we describe how PSIs are used to perform the resections and to adjust the allografts. Finally, we present several clinical cases and their outcomes.

## 2. Materials and Methods

### 2.1. Preoperative Planning for Tumor Resection

Preoperative images of the patients were acquired for diagnoses. Anatomical images were obtained by CT from a Brilliance 40 CT scanner (Philips, the Netherlands; 0.5 mm spacing between slices, 1 mm slice thickness, 120 kV peak voltage, and 99-mA tube current) and by MRI from a 1.5 T NTScan Intera (Philips, the Netherlands, 4 mm spacing between slices, 3 mm slice thickness, 550 ms TR, and 14 ms TE).

Preoperative planning required delineation of each tumor. An MRI series that clearly showed the boundaries of the tumor was selected. The tumor was manually delineated on each slice on which it was visible, using the open-source software ITK-Snap 2.0 (http://www.itksnap.org/) [19]. The obtained delineation, referred to as the tumor volume, was saved for later use.

**Figure 1:**
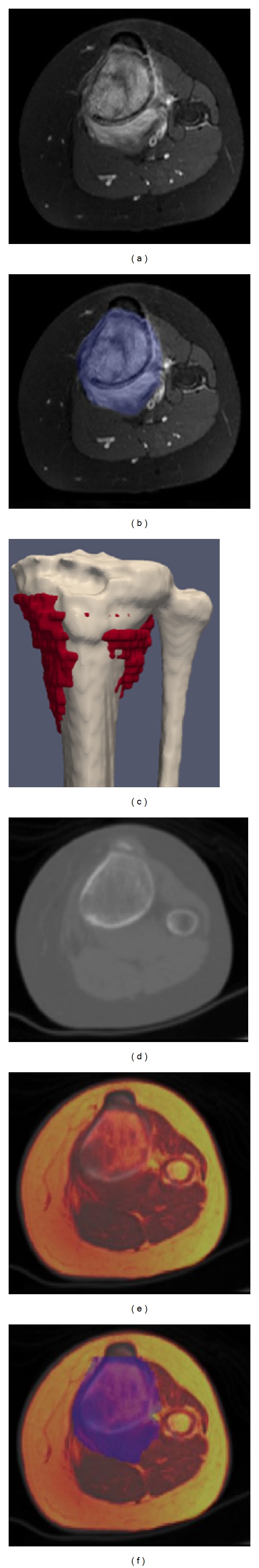
11-year-old patient with tibia metastasis of a femoral osteosarcoma. (a) MRI showing the osteosarcoma invading the proximal tibia. (b) Tumor is delineated in blue on each slice of MRI where it is visible. (d) CT scan. (e) Merging of MRI and CT. (f) MRI-CT merging with the delineation of the tumor. (c) 3D reconstruction of the tibia with the delineated tumor visible (red).

A multimodal registration algorithm was used to merge, simultaneously, the MRI series with the CT images (image fusion) and the tumor volume (Figure 1). 3D models of the bone and the tumor volume were extracted from the 2D slices, and a combined 3D image of the tibia and tumor (red in Figure 1) was obtained. The 3D models were used to position the resection planes (target planes) that represented the trajectories of the saw blade. With the assistance of a haptic device and a specific software program developed in the author's laboratory, the planes were initially brought into contact with the tumor and then translated back with a surgeon-defined security margin of at least 5 mm. This method ensured a controlled safe margin during the surgery. The resection plane data were saved for use in the next two stages of preoperative planning.

### 2.2. Preoperative Planning for Allograft Cutting

To select the best-fitting allograft among the tibial allografts from the local bone bank, CT images of all available tibia allografts were acquired (Somatom Definition AS, Siemens; 0.35-mm slice thickness, 0.7 mm spacing between slices, 120 kV peak voltage, and 99 mA tube current). A monomodal registration was performed between CT scans from the tibia of the patient and the various allografts. The optimal allograft was chosen as the one that best bridged the bone defect created by the resection. Particular attention was paid to the articular surface in the case of an osteochondral allograft. The resection planes defined in the previous stage of preoperative planning were transferred toward the allograft with the registration algorithm (Figure 3). This process ensured that the resection planes were identical for the patient and for the allograft, with a well-matched allograft to fit the bone defect.

### 2.3. Patient-Specific Instruments

Two PSIs were created for each patient: one for the tumor resection and a second one for the allograft cutting. The PSIs were virtually designed with a computer-aided design software package (Blender 2.63.11). Each PSI was endowed with a specific surface that could be fit onto a unique position on the bone surface. The instruments contained small holes that were specifically designed for 2 mm Kirschner wires (K-wires). These K-wires allowed the instrument to be pinned onto the bone surface. A flat surface materialized the resection planes and permitted the clinician to guide the saw blade during the osteotomies. 

**Figure 2:**
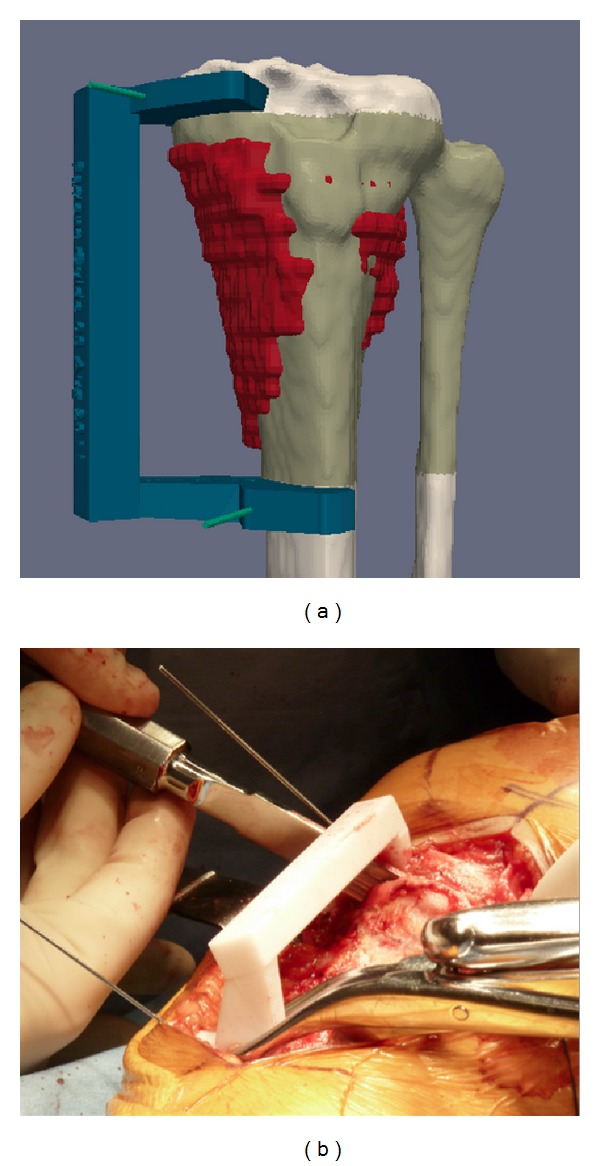
Images from the patient shown in Figure 1. (a) Target resection planes have been designed according to the osteosarcoma (red) with a safe margin. The green zone is included between the two planes. A 5 mm distance (margin) between the plane and tumor was selected to spare epiphysis, whereas a 10 mm margin was chosen distally. K-wires are represented by green cylinders. A PSI was created to guide the saw blade and to respect the planes. (b) PSI has been postitioned at the surface of the exposed tibia, and the surgical saw has been placed on the PSI to follow the target planes.

**Figure 3:**
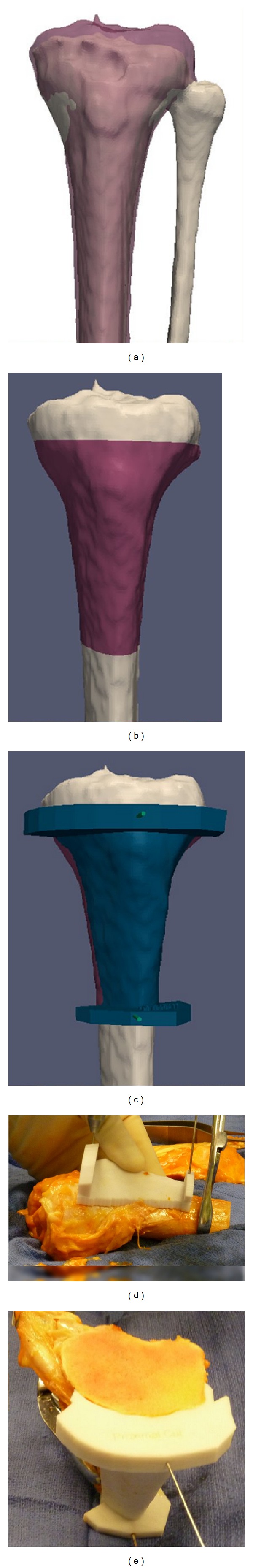
Images from the patient shown in Figures 1 and 2. (a) Merging of the allograft CT and the tibial CT of the patient. (b) Target planes have been transferred to the allograft. The pink zone is the planned cut allograft. (c) A PSI has been virtually created (blue) and is shown pinned to the bone with two K-wires (green cylinders). (d) PSI has been positioned on the surface of the tibial allograft. (e) Allograft after cutting with the saw.

The PSIs were produced by rapid prototyping with Selective Laser Sintering (SLS) technology in a biocompatible material (Polyamide, Figure 2). This additive manufacturing technology consists of building a 3D object by adding material layer by layer. A laser draws a 2D shape on the surface of a powder bed, locally fusing the powder to create a solid section. A new layer of powder is spread on top of the surface, and the process is repeated until the entire object is built.

### 2.4. Patient Series (Table 1)

Four patients were operated on for tibial bone sarcoma resection and allograft reconstruction with PSIs. Two patients presented with primary tibia sarcomas (one with osteosarcoma and one with Ewing's sarcoma), one patient had a local recurrence of tibial osteosarcoma, and one patient had a tibial metastasis from a contralateral femoral osteosarcoma. The ages at operation ranged from 9 years and 9 months to 18 years and 2 months. Imaging assessment consisted of plain radiograph, CT, MRI, and positron emission tomography (PET)-CT with fluorodeoxyglucose-18. The latter revealed no distant metastases. Neurological deficits and systemic symptoms, such as fever or weight loss, were absent. The diagnosis was confirmed by an incisional biopsy and histological evaluation of the tissue. Patients received multiagent, neoadjuvant, and adjuvant chemotherapy, according to the Euramos, Euro-Ewing, OS2005, and OSII-TTP protocols.

### 2.5. Assisted Surgery

The patients were placed in decubitus. A medial-tibial approach was used in three patients and a lateral approach in the last patient (because the biopsy had been performed laterally). The open-surgical biopsy tract was excised with the tumor, as an ellipse. A progressive soft-tissue dissection was performed to isolate the tumor with the planned margin and with the preservation of the anterior tibial tuberosity. Both PSIs (one for the patient and one for the allograft) were sterilized by standard autoclaving the day before the surgery. The resection PSI was positioned on the tibia and rigidly fixed with two K-wires at proximal and distal locations (Figure 2). A safe margin of 5 mm was chosen (3.5 mm after resection because of material loss due to saw blade cutting, i.e., the kerf). Proximal and distal osteotomies were performed with a surgical saw that was guided by the PSI. The tibial resection lengths ranged from 8 to 16.4 cm. 

Extemporaneous biopsies were taken from both the distal and proximal sites in the tibia to ensure the adequacy of the margins. After the pathologic confirmation of the safe margin, reconstruction was undertaken. During tumor excision, a second surgeon prepared the allograft to reconstruct the resulting surgical defect. After soft-tissue dissection, the allograft-specific instrument was pinned onto the graft. The instrument was created such that the osteotomy planes of the graft were receded 1.5 mm to compensate for the kerf.

**Figure 4:**
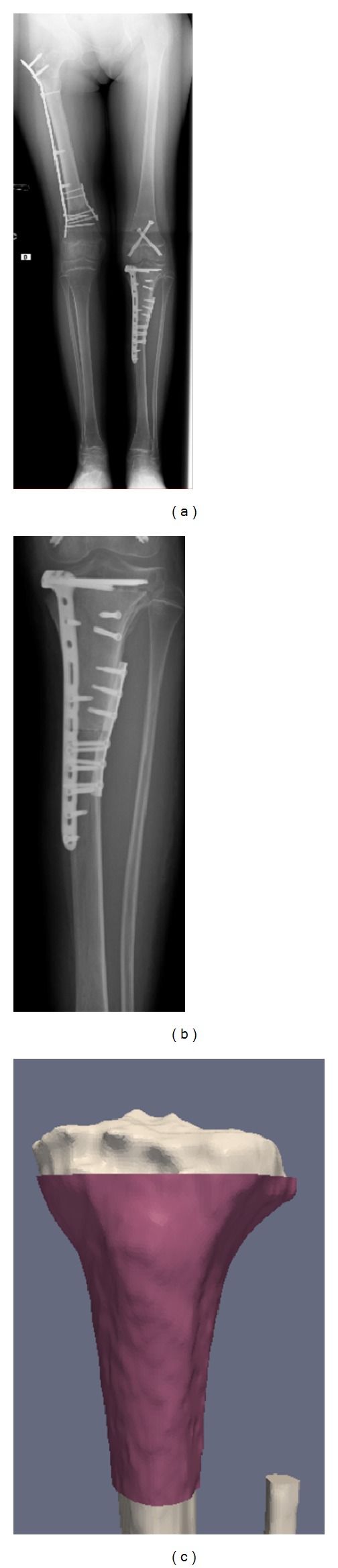
Images from the patient shown in Figures 1, 2, and 3. (a) Full-length standing radiograph showing the final result. (b) Magnified view of the reconstruction site: the intercalary allograft is inserted between the spared epiphysis and diaphysis. Osteosynthesis is performed with two plates. (c) Virtual simulation of reconstruction with allograft (pink) inserted into the patient's tibia (white).

The adjusted allograft was placed on the bone defect, and osteosynthesis was performed with an NCB titanium 9-hole plate in three cases, and with two plates (an L-plate and a 6-hole plate) in one case (Figure 4). In two patients, a contralateral proximal tibia epiphysiodesis was performed at the same time to avoid a consecutive progressive leg-length discrepancy. A brace was applied for all patients in the postoperative period.

## 3. Results

The mean total surgical time was 250 minutes from the time of skin incision to the end of skin closure. Preoperative preparation, including general anesthesia, epidural catheter insertion, patient positioning, and draping, lasted a mean of  73.5 minutes.

One patient developed transient common fibular nerve palsy, which was confirmed by electromyography. Skin necrosis and wound dehiscence occurred in the patient with the osteoarticular allograft at one postoperative month, necessitating a lateral gastrocnemius flap. The allograft was ultimately found to be infected by *Enterococcus faecium* and *Pseudomonas* and was explanted 3 months after the reconstruction. An antibiotic-impregnated cement spacer was inserted, and a new reconstruction was performed 4 months later with a new PSI to guide the allograft cutting. 

Histological examination of the removed sarcoma confirmed osteosarcoma in three patients and Ewing's sarcoma in one patient. All resection margins appeared to be tumor-free. Postoperative radiograph, CT scan, and MRI results revealed satisfactory host-graft contact and no evidence of recurrent disease. The explanted, infected allograft was not consolidated with the host tissue. For the other three patients, radiological union was obtained at the graft-host junction at 4, 9, and 12 months. Partial weight bearing was allowed after 6 weeks and full weight bearing after 3 months, except for one patient for whom partial weight bearing was allowed immediately. 

## 4. Discussion

This paper reports a novel method of bone sarcoma surgery that is supported by the use of PSIs. The PSI assistance was used not only for tumor resection but also for massive allograft cutting, allowing for optimal reconstruction. This technique was applied to 4 patients.

**Figure 5:**
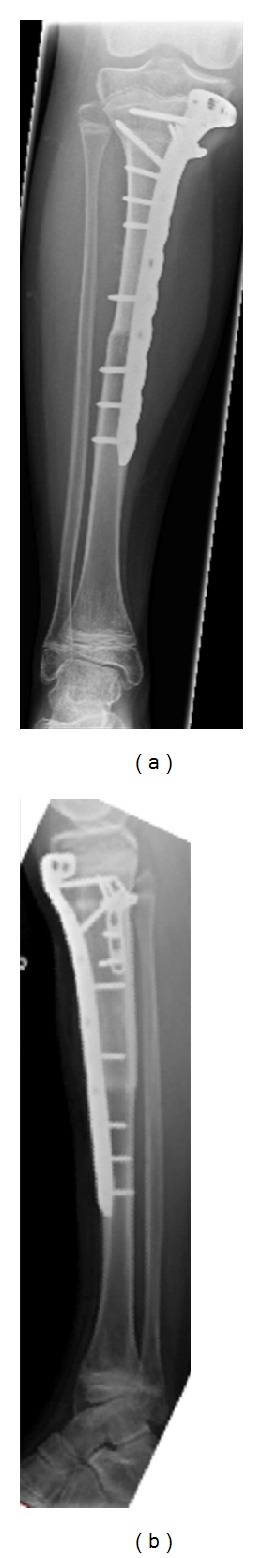
12-year-old boy with EWS sarcoma of the proximal tibia. Result of the reconstruction with preservation of the growth plate: radiographic anteroposterior and lateral view.

In surgical oncology, obtaining a wide margin during a tumor resection is crucial to avoid local recurrence. However, limb-salvage surgery requires the preservation of a functioning limb at the expense of obtaining safe margins [3]. Accurate preoperative localization of the tumor provides full control over the safe margins, and PSIs improve the accuracy of the resection during the surgery. The combination of these techniques allows resection with adequate but minimal safe margins, thus preventing unnecessary resection and preserving, when needed, articular cartilage in young patients. In one of the patients presented here, the target margins were defined at 3.5 mm, which allowed for the preservation of the growth plate (Figure 5). This outcome would not have been possible without the assistance of PSIs.

The literature reports discrepant results for tibia allografting reconstruction, ranging from excellent outcomes with full incorporation and osteointegration of the allograft and durable joint function to a high rate of failure and complications, including accelerated and advanced arthritis, fractures, nonunion, and infection [20]. The PSI technique allows the allograft to be cut with a high degree of accuracy, producing a transplant of the optimal shape to bridge the bone defect. Moreover, the allograft can be adjusted simultaneously (by another surgeon) or prior to the tumor resection, thereby decreasing the operating time and improving patient safety. The precise tumor and allograft cuttings obtained by PSIs yield strong contact at host-graft junctions, resulting in a stable osteosynthesis. The mechanical stability of the graft facilitates improved and more rapid healing and bone fusion due to the increased growth of blood vessels into the graft [21].

The production of PSIs requires medical images to be sent to an engineer who performs the preoperative planning. This process presents a challenge because it is crucial to maintain the security of the medical data of the patient. In addition, an open communication between the surgeon and the engineer is important. Furthermore, the engineer must have a strong clinical background to understand the medical context and the prerequisites of the PSI that will be generated. The PSI must be localized to a unique site on the bone surface that will be exposed by the surgical approach without adding unnecessary surgical approaches, skin incisions, or dissection. Surgeons will be asked to anticipate the constraints of the surgery (e.g., surgical approach, access to bone surface, and presence of soft tissue). The engineer must respect these clinical data and determine a trade-off between the invasiveness of the PSI and its stability on the bone surface. In our series of patients, accurate positioning of the instruments was easily achieved for each case, and the instruments were stable.

## 5. Conclusions

The techniques described in this paper may help to improve patient safety and surgical accuracy for the resection of bone sarcomas of the tibia and for the accompanying reconstruction at these sites. In addition, these techniques may potentially benefit surgery for bone sarcomas at other sites such as the pelvis.

## Figures and Tables

**Table 1:** Summary of 4 cases of tibial resection with PSI.

	Case 1	Case 2	Case 3	Case 4
Patient information				
Age (y)	18.2	12.5	11.2	9.8
Sex	M	M	F	M
Sarcoma type	OS (local occurrence)	EWS (primary)	OS (tibial metastasis of a femoral OS)	OS (primary)

Resection				
Type	Intercalary	Intercalary	Intercalary	Osteoarticular
Length (cm)	8.0	11.8	8.2	16.4
Preservation	Preservation of epiphysis	Preservation of proximal tibial growth plate	Preservation of epiphysis; sacrifice of growth plate	Epiphysiodesis of contralateral proximal tibia

Results				
Local complications	None	Transient common fibular nerve palsy	None	Sepsis requiring allograft replacement
Time to host-allograft junction union (mo)	4	9	12	None
Followup (mo)	17	19	14	10
Final patient outcome	Excellent; walking without assistance	Excellent; walking without assistance	Excellent; walking without assistance	Poor after initial procedure; good final result; walking with a brace

M: male; F: female; OS: osteosarcoma; EWS: Ewing's sarcoma.
